# Optimizing Hard and Soft‐Tissue Esthetics With Anterior Cantilever Zirconia Ceramic Resin‐Bonded Fixed Dental Prostheses

**DOI:** 10.1111/jerd.70025

**Published:** 2025-08-27

**Authors:** Markus B. Blatz, Tony Rotondo, Szabi Hant, Lea S. Prott

**Affiliations:** ^1^ Department of Preventive and Restorative Sciences School of Dental Medicine, University of Pennsylvania Philadelphia Pennsylvania USA; ^2^ Private Practice Brisbane Australia; ^3^ Private Practice Perth Australia; ^4^ Department of Prosthodontics, Medical Faculty and University Hospital Düsseldorf Heinrich‐Heine‐University Düsseldorf Germany

**Keywords:** ceramic bonding, congenitally missing teeth, ovate pontic design, resin‐bonded fixed dental prosthesis, ridge augmentation, zirconia cantilever fixed dental prosthesis

## Abstract

**Objective:**

The replacement of missing maxillary lateral incisors poses both functional and esthetic challenges, not only from a restorative but also from a periodontal aspect. This case report presents a step‐by‐step protocol for ideal hard and soft‐tissue esthetics with cantilever zirconia ceramic resin‐bonded fixed dental prostheses (RBFDPs).

**Clinical Considerations:**

A 20‐year‐old female patient presented with congenitally missing maxillary lateral incisors, seeking treatment to enhance the esthetics of her smile. Due to the age and ongoing skeletal development, implant placement was deferred. Treatment involved the extraction of a retained deciduous tooth, soft tissue augmentation with a subepithelial connective tissue graft, and the placement of zirconia ceramic single‐retainer RBFDPs. The pontic design and tissue contouring were carefully managed with a removable partial denture to achieve a natural emergence profile and long‐term stability.

**Conclusion:**

The combination of RBFDPs and soft tissue augmentation offers a highly esthetic and minimally invasive treatment alternative for replacing missing anterior teeth. Proper case selection, bonding technique, and soft tissue management are critical for long‐term success.

**Clinical Significance:**

This report highlights the clinical efficacy of zirconia RBFDPs as a conservative alternative to implants in young patients and demonstrates the importance of soft tissue management for optimal esthetic integration.

## 1 Introduction

Congenitally missing teeth in the anterior region often demand an immediate solution with either a temporary or permanent restoration to address esthetic and functional concerns [1, 2]. Hypodontia, characterized by the absence of permanent teeth, affects approximately 2.5% to 6.9% of the population, with maxillary lateral incisors being the most commonly missing anterior teeth, often bilaterally [3, 4]. Treatment options for these cases include orthodontic space closure, autotransplantation of deciduous or permanent teeth, implant‐supported restorations, conventional fixed dental prostheses (FDPs), and cantilever resin‐bonded fixed dental prostheses (RBFDPs) [1, 5, 6].

Although implant therapy is often the preferred treatment, it may not always be the optimal option. Factors such as the patient's age, limited space between the adjacent teeth/roots, inferior hard and soft tissue quality, financial limitations, or simply the patient's preferences may necessitate alternative solutions [7, 8]. RBFDPs provide a minimally invasive alternative to both implant therapy and conventional crown‐retained prosthetics with excellent outcomes in terms of durability, esthetics, function, and overall patient satisfaction [5, 9]. They are especially well suited for replacing lateral incisors, where the retaining teeth are robust and the pontic spaces are small. Driven by increasing esthetic demands, all‐ceramic RBFDPs have gained popularity [10], with reported high survival rates of 100% after 10 years [11] and 81.8% after 18 years [12]. While obviously rare, the primary cause of failure is debonding, followed by framework fracture [13]. The use of zirconia for RBFDP fabrication has reduced the incidence of framework fractures [5, 14]. While debonding remains the most common complication [15], a debonded restoration can often be easily rebonded. In comparison to the often severe biological and technical failures associated with implant‐supported restorations and conventional FDPs, complications with RBFDPs are rarely catastrophic. In addition, more invasive treatment options can usually still be applied later, and, by delaying those, patients may ultimately be able to retain their natural teeth longer.

For long‐term bonding success, airborne particle abrasion in combination with a 10‐methacryloyloxydecyl dihydrogen phosphate (MDP) primer and composite resin cement has proven to be the most reliable protocol [16, 17]. Bonding under rubber dam isolation may further enhance the clinical survival rate [18]. To date, RBFDPs with single retainer designs are the preferred option, as they reduce the risks of caries, debonding, and fracture compared to two‐retainer counterparts [19].

Post extraction alveolar ridge deformities can pose challenges to esthetic rehabilitations. Conventional restorative approaches, such as the extension of the intaglio pontic portion, often result in esthetic and functional concerns. These include disproportionate restoration dimensions, papilla loss, open interdental “black triangle” spaces, food impaction beneath the pontic, as well as phonetic difficulties [20]. For this reason, augmentation techniques to correct soft tissue defects have become of interest [21].

This case report presents a surgical and prosthetic approach for achieving optimal esthetics in the replacement of missing maxillary lateral incisors with cantilever zirconia RBFDPs. Ideal pontic site development with an interim removable partial denture immediately after extraction and after augmentation with a subepithelial connective tissue graft (SCTG) is demonstrated in a step‐by‐step manner.

## 2 Clinical Report

**FIGURE 1:**
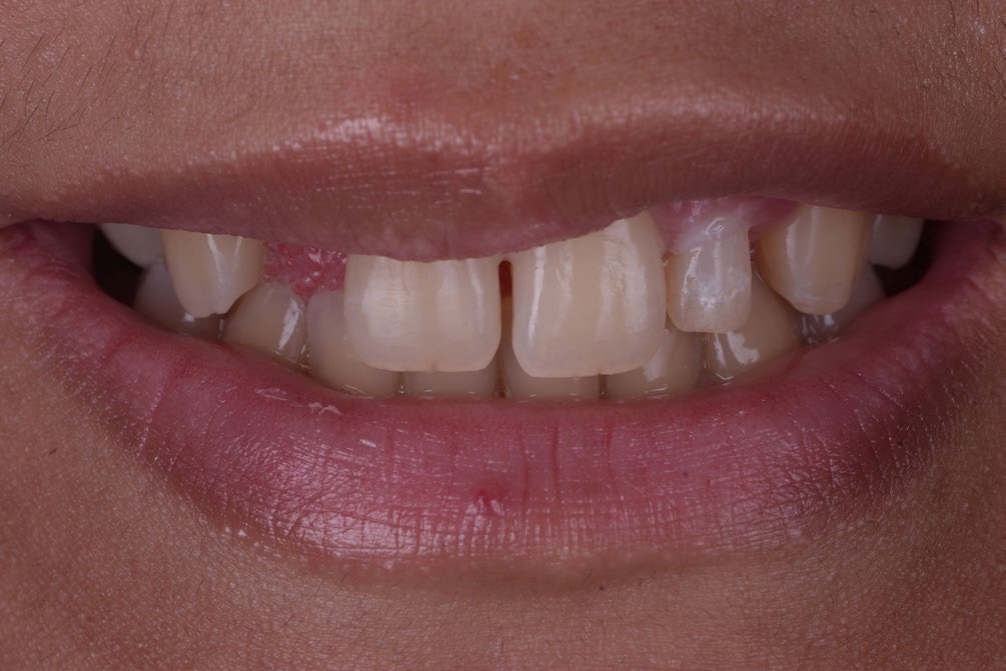
Initial situation extraoral.

**FIGURE 2:**
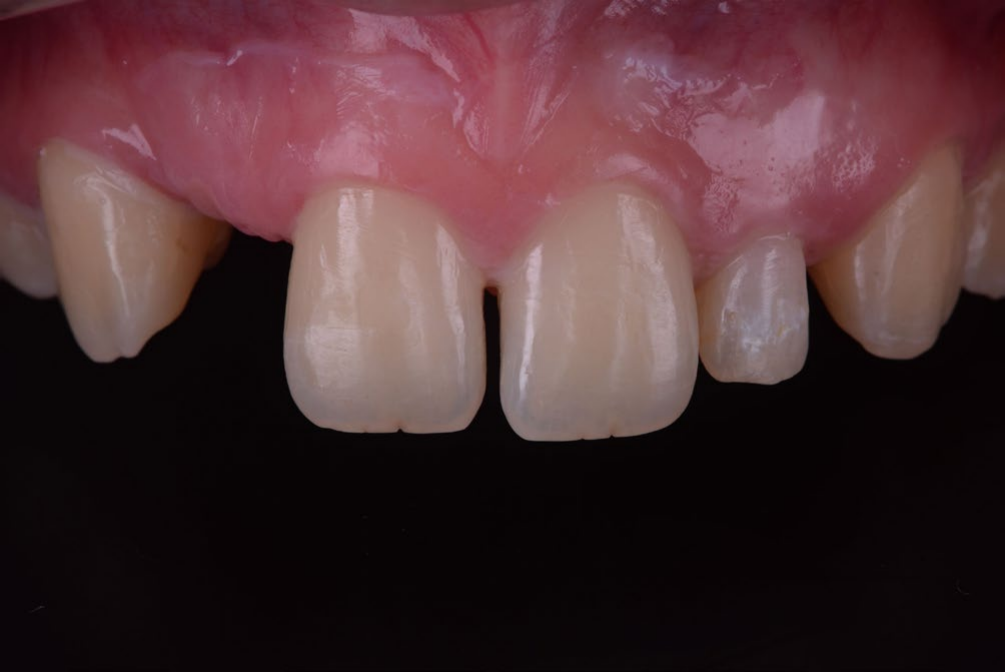
Initial situation intraoral.

A 20‐year‐old female patient presented after completing orthodontic treatment to create adequate space for her missing bilateral maxillary lateral incisors. The left deciduous tooth was still retained (Figures 1 and 2). The patient expressed a desire for a fixed, esthetically pleasing treatment solution. Clinical evaluation revealed vital teeth and stable periodontal health. However, a horizontal and vertical loss of ridge structure (Seibert Class III) [22] was observed in the area of the missing right lateral incisor. The patient was informed about various treatment options, including implant therapy, conventional FDPs, and RBFDPs. Since implant placement was considered unsuitable at her age, RBFDPs presented a viable alternative treatment for replacing the maxillary lateral incisors. After careful consideration, the patient chose the option of cantilever zirconia RBFDPs with soft tissue augmentation beforehand.

**FIGURE 3:**
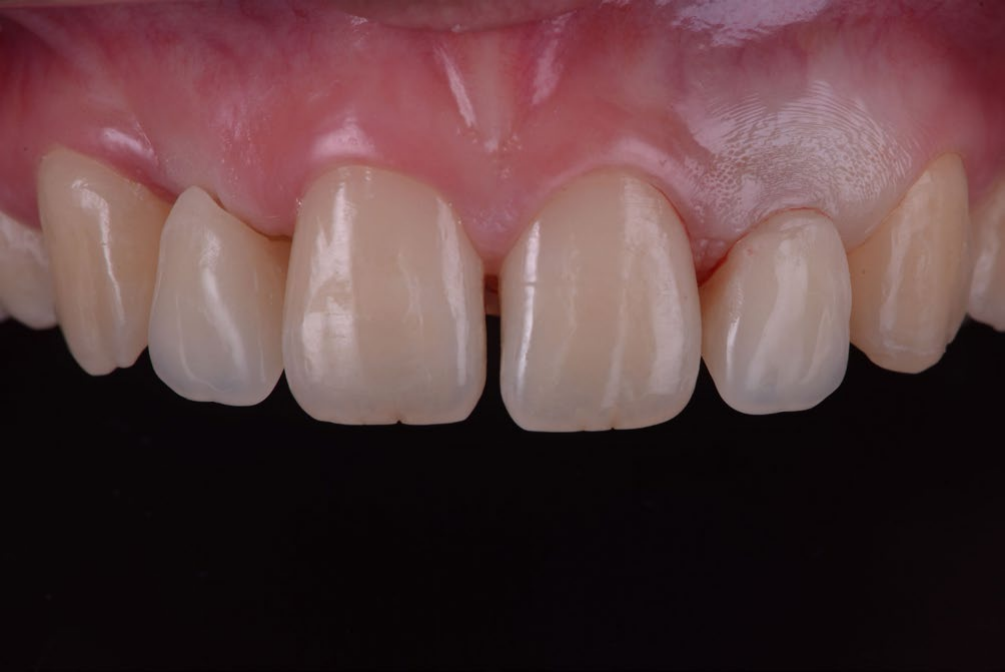
Intraoral frontal view with immediate removable partial denture after insertion.

The deciduous tooth was extracted, and an immediate removable partial denture was placed to restore function and esthetics by replacing the missing lateral incisors. Once hemostasis was achieved at the extraction site, the pontic was meticulously adjusted using flowable composite resin, with 3–4 mm of its apical portion extending into the socket (Figure 3). This immediate modified ovate pontic technique supports both the underlying bone and soft tissue previously supported by the extracted tooth root while applying gentle, controlled pressure to the extraction site [23]. The applied pressure supports and maintains the interdental papilla and creates a natural emergence profile, enhancing the illusion of a natural tooth seamlessly emerging from the gingiva [24]. Additional layers of flowable composite were successively applied to further refine the site, specifically to reposition the gingival crest in a more labial and apical direction.

**FIGURE 4:**
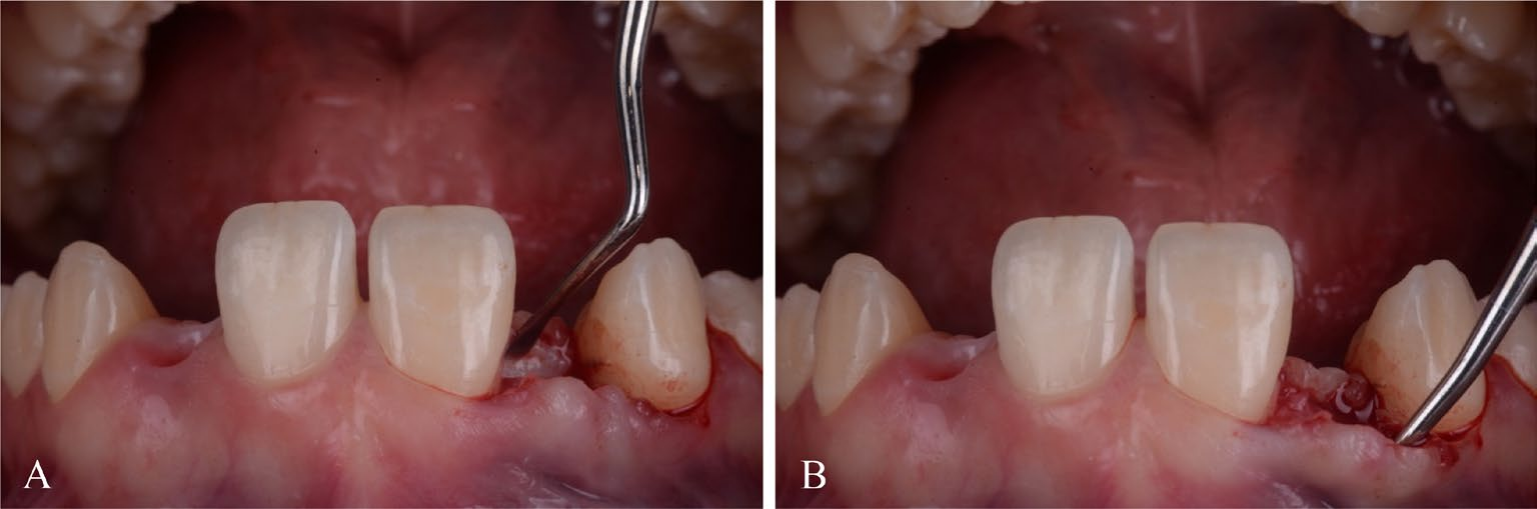
Supraperiosteal (A) palatal and (B) labial tunnels were dissected in the edentulous ridge area to create a recipient bed for the subepithelial connective tissue graft.

**FIGURE 5:**
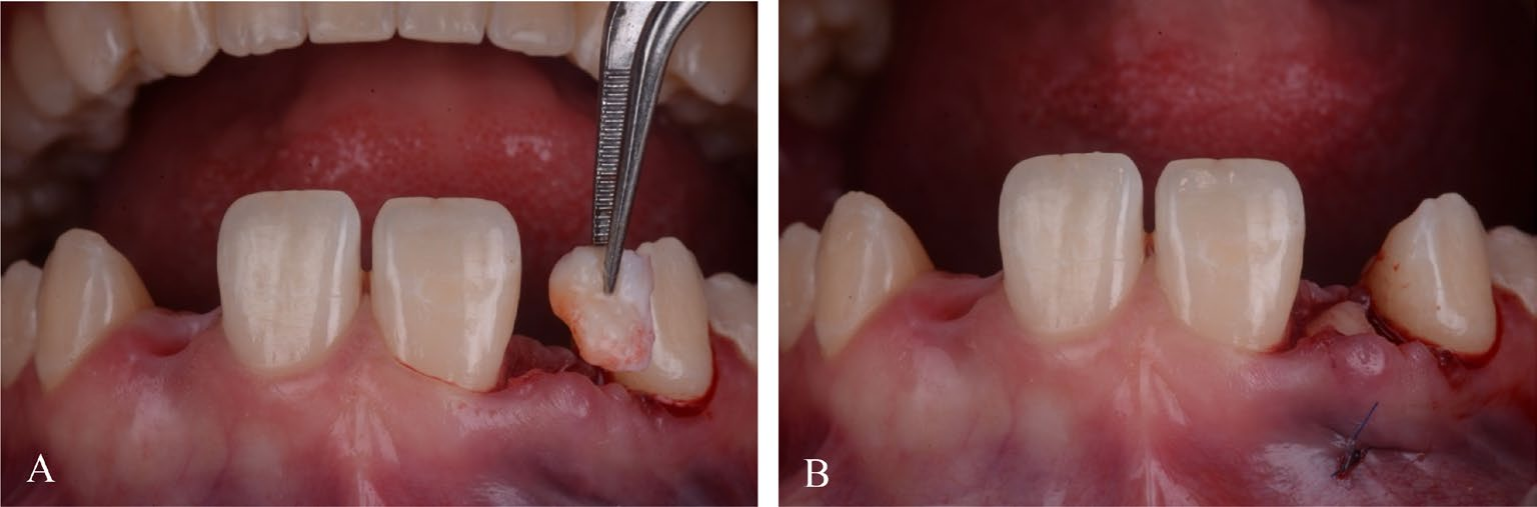
(A) The subepithelial connective tissue graft was placed into the labial tunnel of the prepared recipient bed and (B) was fixed with a suture at the apical end.

**FIGURE 6:**
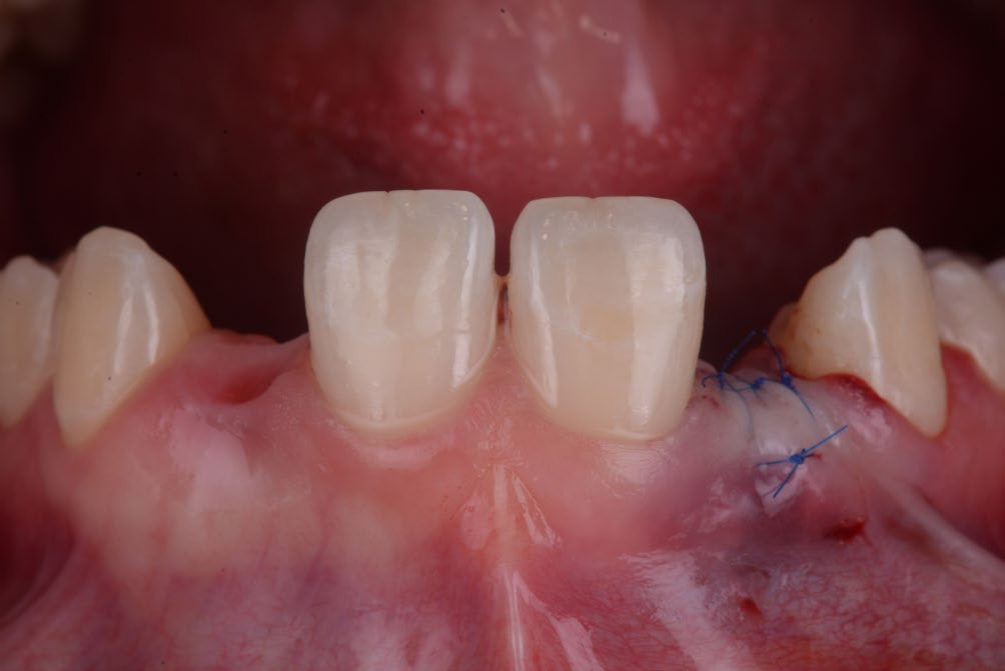
Situation after soft tissue augmentation.

**FIGURE 7:**
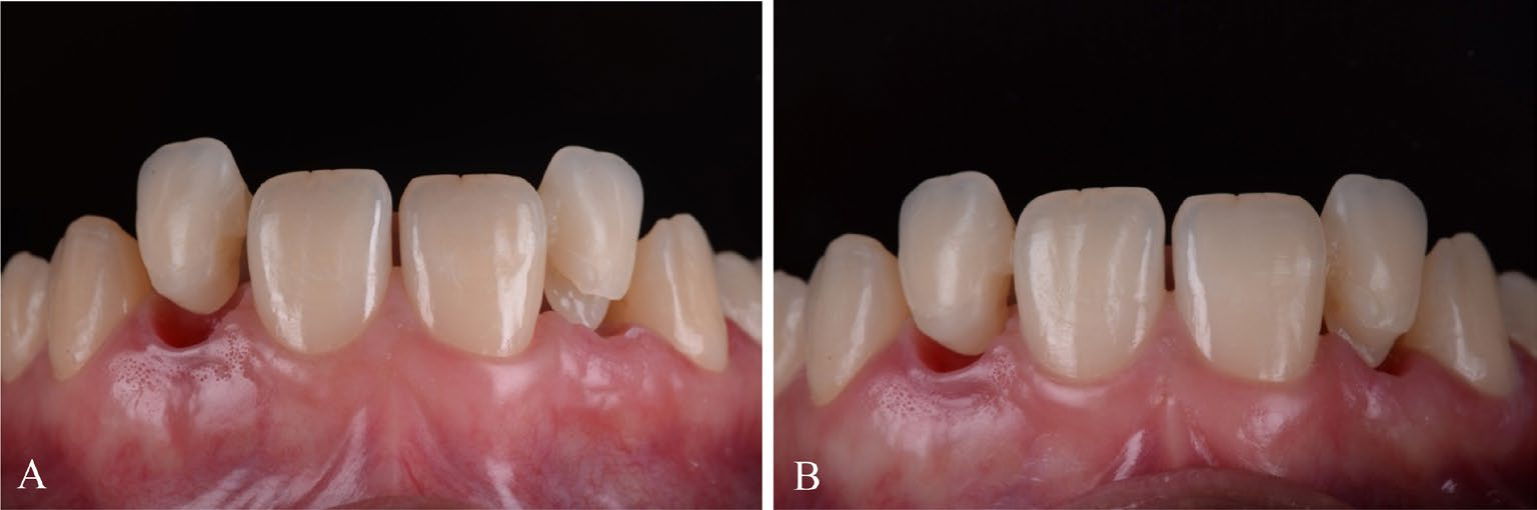
Partially seated removable partial denture. (A) After a two‐month waiting period, the pontic side in the first quadrant was developed and (B) progressively refined with additional applications of flowable composite over time.

**FIGURE 8:**
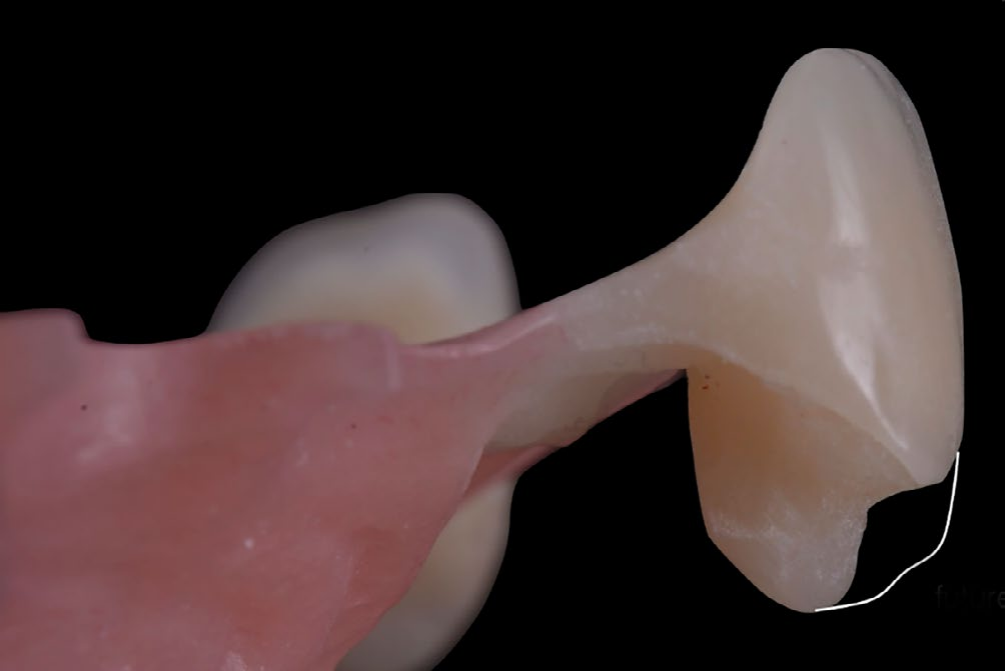
Over time, the illustrated area was gradually filled with flowable composite.

**FIGURE 9:**
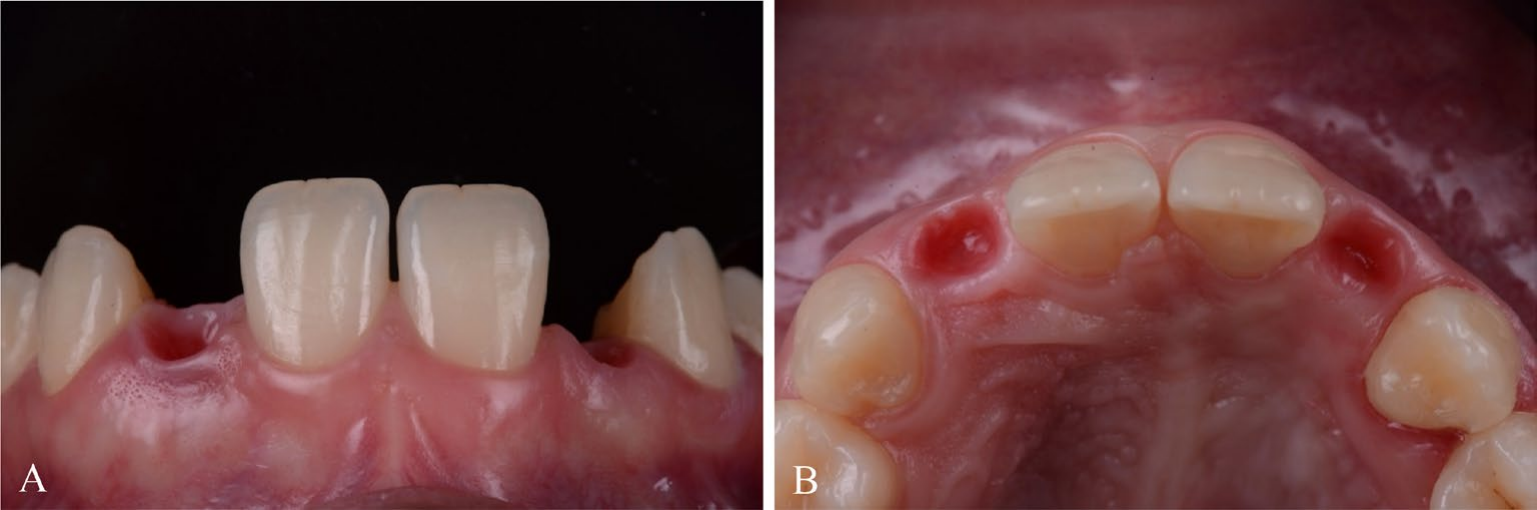
Soft tissue management results. Intraoral (A) frontal and (B) occlusal view after adjustments of pontics.

A SCTG was placed to improve soft tissue volume and contour in the area of the missing right lateral incisor [25]. A partial‐thickness flap was prepared from the incision line with supraperiosteal labial and palatal tunnels (pouch technique), allowing a closed adaptation of the graft to the recipient side (Figure 4A,B). The SCTG was harvested from the maxillary tuberosity. Two parallel incisors, approximately 2 mm apart, were made distal to the second molar, followed by a releasing incision at the base of the graft. After harvesting the graft, the epithelium was removed to obtain the SCTG. The SCTG was then placed into the labial tunnel of the prepared recipient bed (Figure 5A) and fixed with a suture at the apical end of the tunnel (Figure 5B). Horizontal mattress sutures were used to guide the graft into position and to secure the SCTG on the palatal side. Interrupted sutures were placed at sulcular margins (Figure 6). Sutures were removed 10 days post‐surgery and the provisional pontics were relined to the desired shape. Site development resumed at 8 weeks, starting with a spade‐like perforation of the residual ridge, facilitated by a mid‐crestal incision. The site was then progressively refined with additional applications of flowable composite to the denture pontic, each applied at two‐week intervals (Figures 7A,B and 8). Figure 9A,B depicts the final result of the soft tissue management.

**FIGURE 10:**
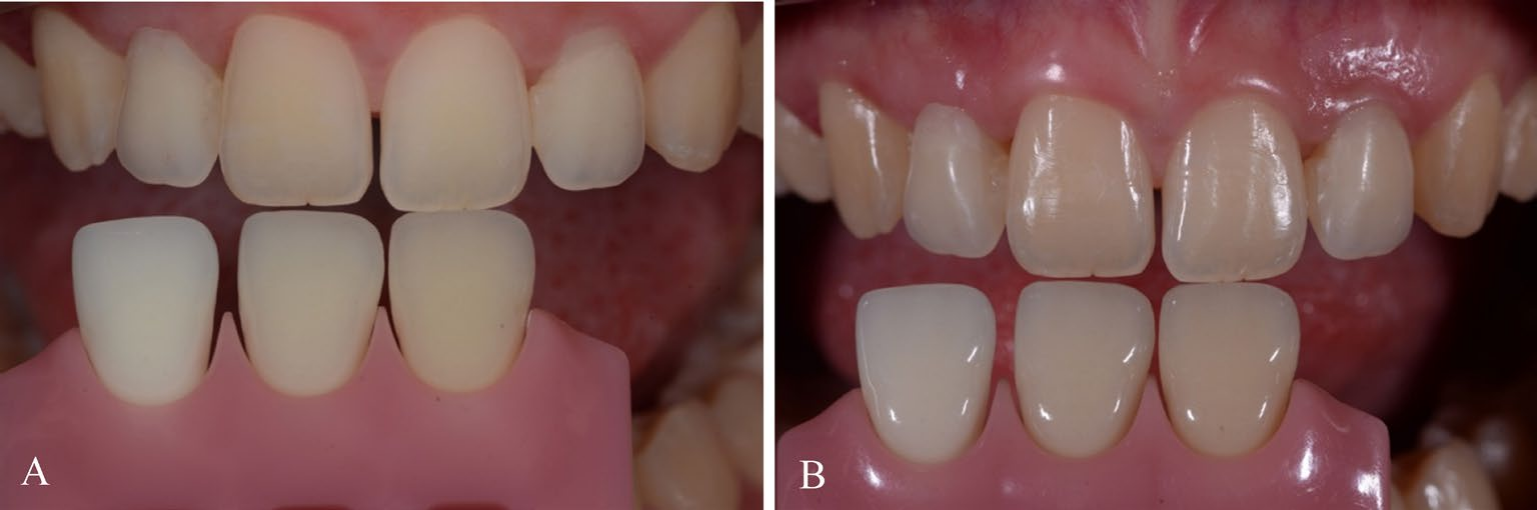
Shade selection. Pictures were taken (A) with and (B) without polarized filter.

Minimally invasive preparation of the canines was conducted with fine graded diamond burs. A light chamfer was created at the gingival crest, and a margin was positioned 2–3 mm from the incisal edge. Proximal preparation was also performed to parallel the proximal walls and increase the surface area. Finally, a central depression was created using a large round bur to facilitate the accurate positioning of the definitive RBFDP. A polysiloxane impression (Imprint 4, 3MEspe, St. Paul, USA) was made using a double‐mix technique. At the beginning of the appointment, the shade was determined with a shade guide (Ivoclar AD shade guide, Ivoclar Vivadent, Schaan, Liechtenstein). Photographs were taken with and without polarized filter (Intra.polar, Perth, Australia) (Figure 10A,B). Restorations were milled out of 3 mol% yttria‐stabilized tetragonal zirconia polycrystal (3Y‐TZP, ZirCAD LT, Ivoclar Vivadent), with the labial surface veneered with feldspathic ceramic (Ivoclar Vivadent). Minimal framework dimensions were verified, with a connector width of 2 mm, a height of 3 mm, and retainer wings measuring a minimum thickness of 0.7 mm.

**FIGURE 11:**
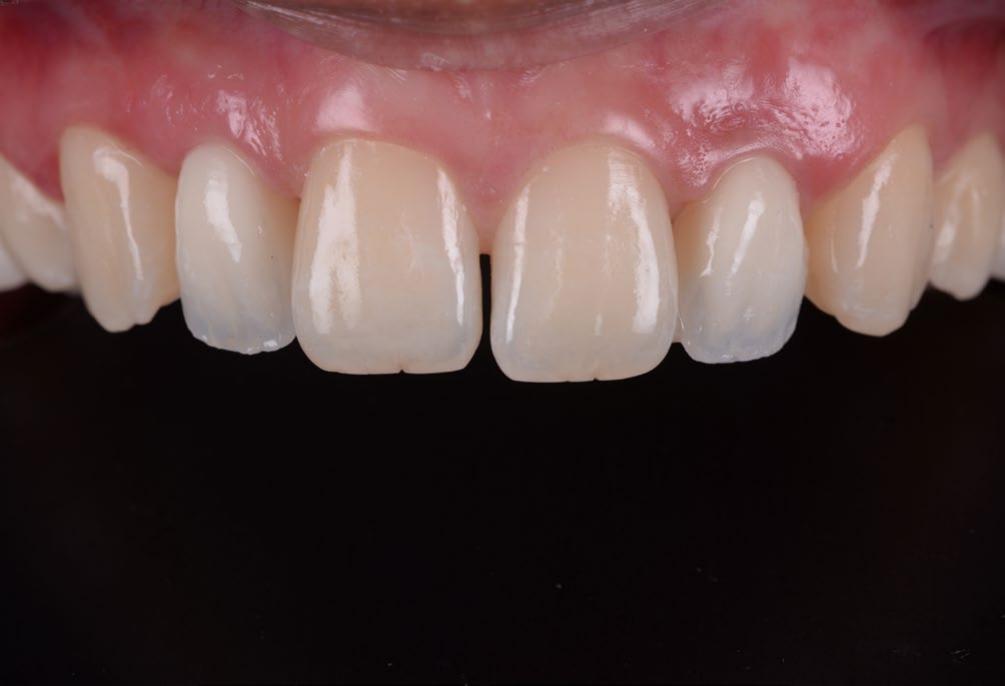
Try‐in of final restorations.

**FIGURE 12:**
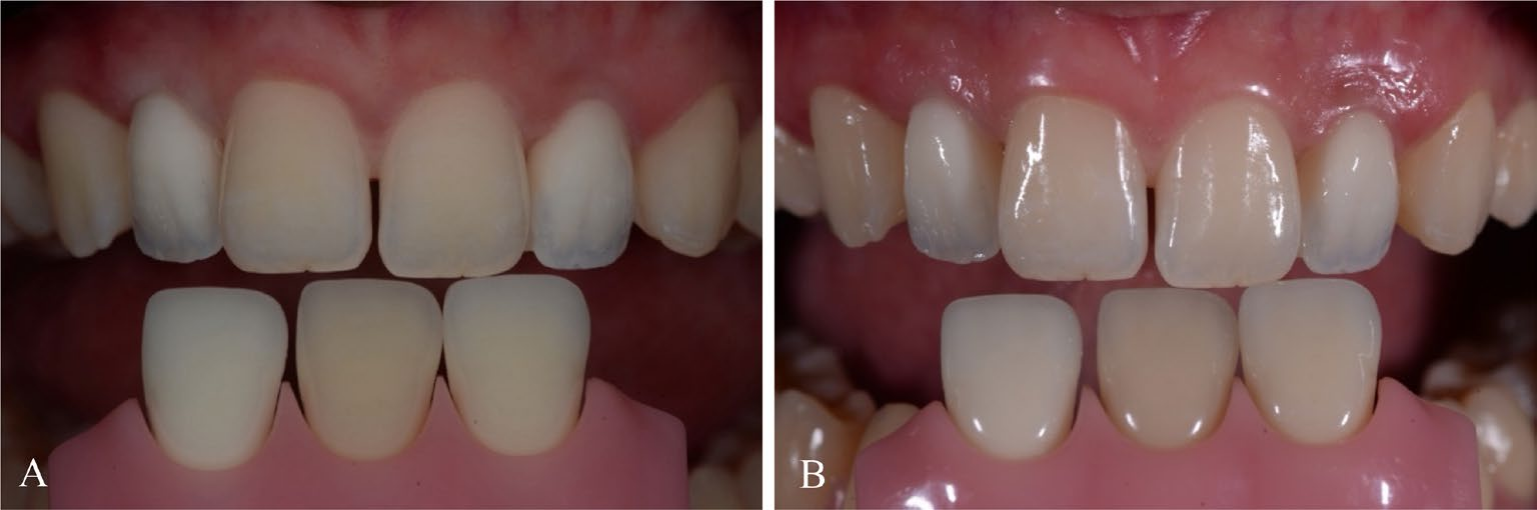
Shade adaptation during try‐in appointment. Pictures were taken (A) with and (B) without polarized filter.

The partially completed restorations were tried in to assess esthetics, phonetics, as well as the pontics' relationship with the soft tissue (Figure 11). Intraoral photographs were taken again with the shade guide and a camera with and without polarized filter (Figure 12A,B).

**FIGURE 13:**
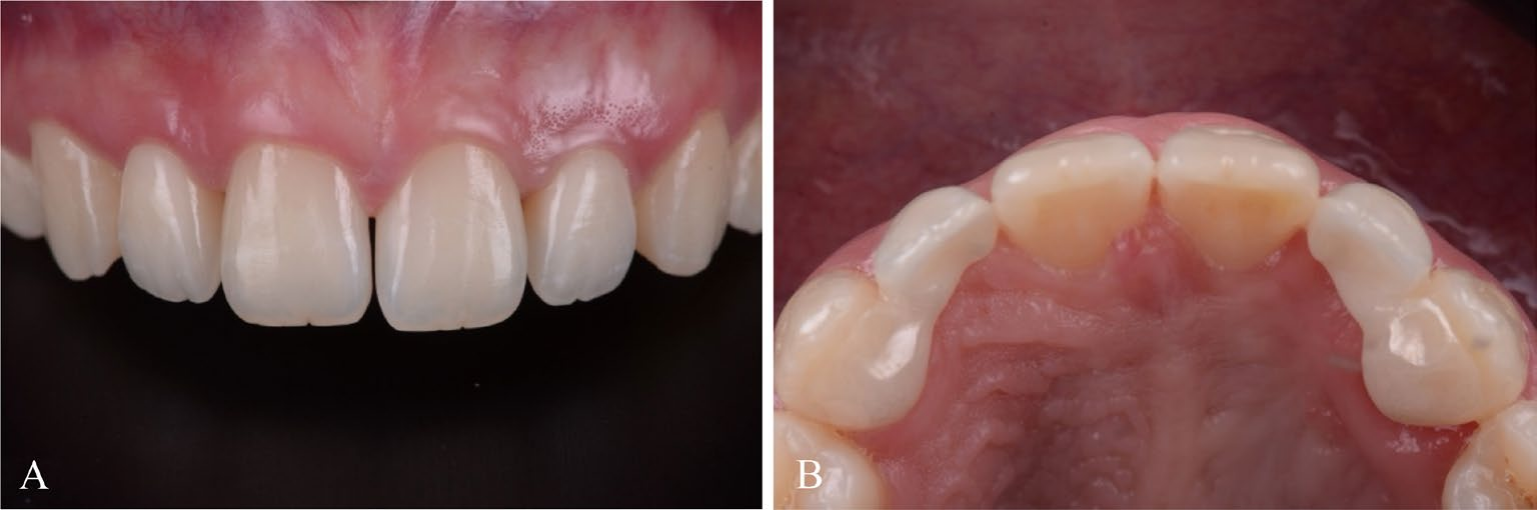
Intraoral (A) frontal and (B) occlusal view of maxilla with final restorations after cementation.

After try in, the restorations were cleaned in an ultrasonic cleaning bath in alcohol. After drying, the intaglio surfaces of the retainers were airborne particle‐abraded (50 μm Al_2_O_3_ particles at 1 bar pressure for 5 s), while the enamel was etched with 37% phosphoric acid for 30 s. A ceramic primer containing functional phosphate monomers (Monobond Plus, Ivoclar Vivadent) was applied for 60 s. RBFDPs were inserted using a composite‐based resin cement (Multilink, Ivoclar Vivadent). Intraoral photographs of final restorations were taken afterward (Figure 13A,B).

## 3 Discussion

Cantilever zirconia ceramic RBFDPs have demonstrated excellent clinical long‐term performance, with a 15‐year survival rate of 97.7% for the replacement of maxillary lateral incisors [26]. In comparison, alternative treatments, such as implant‐supported single crowns (93.3%) and conventional FDPs (82.8%) have shown lower survival rates in the anterior maxilla after 15 years [27]. The success of RBFDPs depends on several factors. These include appropriate case selection [28], RBFDP design [14], ceramic material [9], bonding technique [16], operator skills [29], and follow‐up care [30]. Compared to implant therapy, RBFDPs offer the advantage of being a viable option for young patients with the potential for continuous growth as well as cases with limited space (< 7 mm), where implant placement may be challenging or impossible [5]. Ideal conditions for RBFDPs are caries‐free, well‐aligned abutment teeth that provide sufficient enamel for bonding. Additionally, good oral hygiene, patient compliance, and the absence of parafunctional habits and periodontitis are desirable for long‐term success [14, 31]. The above‐mentioned studies employed a strict patient selection process, including only individuals with caries‐free abutment teeth (or minor defects), no signs of periodontitis, and appropriate occlusion. Additionally, the patients exhibited a high level of compliance with their yearly recall appointments. Consequently, the findings are specific to this patient group and should be interpreted with caution when applied to other patients [30]. Appropriate patient selection seems to be key for the long‐term success of RBFDPs [31].

Due to their superior flexural strength, RBFDPs made of zirconia can be designed with significantly smaller connector and retainer wing dimensions than with other ceramic materials and exhibit a lower incidence of framework fractures [14]. In most cases, debonded RBFDPs can be successfully rebonded without further complications, reducing the overall failure probability of zirconia RBFDPs [32]. Conventional hydrofluoric acid etching‐silane treatment was shown to be ineffective for the bonding of zirconia. Instead, air‐particle abrasion is recommended to enhance mechanical retention, increase free surface energy, and decontaminate the bonding surface [33, 34] Previous reviews have indicated that achieving long‐term durable bonding to zirconia ceramics under humid and high‐stress environments is best accomplished by combining air‐particle abrasion at moderate pressure with the application of MDP‐containing luting resins [32, 35]. The APC Concept, including (A) air‐particle abrasion, (P) MDP‐primer, and (C) adhesive composite resin, has proven to be most effective for achieving strong and durable bonds to zirconia restorations [34, 36]. A previously published review reported that air‐particle abrasion with 50 μm alumina particles was the most commonly used method. Using a zirconia primer containing 10‐MDP was reported to be essential for a reliable bond, while the choice of the cement was not critical for bonding success [36]. Despite extensive laboratory and clinical evidence demonstrating excellent long‐term survival rates, reported as 97.3% for maxillary and mandibular incisors after 15 years [26], many clinicians still question the long‐term stability of zirconia bonding. Novel surface treatment methods, such as high‐concentration acid etching, are being developed but have so far failed to demonstrate significant improvements over the well‐documented APC Concept [37]. Others prefer silica‐based ceramics for RBFDPs because of their superior bonding potential. Silica‐based ceramics are certainly an option but require significantly larger connector and retainer wing dimensions due to their lower fracture strength [26]. Indeed, the quality of resin bonds to silica‐based ceramics differs from those to zirconia ceramics, even though actual bond strength values are similar [38]. There is a common misunderstanding in the argument to prefer silica‐based ceramics due to “better bondability”. Tooth‐ceramic bonding involves two distinctly different bonding interfaces: one to the tooth structure and one to the ceramic. When the bond strength to the ceramic is higher than the one to the tooth, it does not matter how much better it is: the bonding interface to the tooth becomes the weak link and, therefore, more likely to be the cause of failure than the one at the ceramic interface. Bond strengths to dentin and enamel are typically lower than those achieved to zirconia with the APC technique, especially in a clinical setting without rubber dam isolation [39]. Therefore, “better bondability” to the ceramic has little relevance to the clinical success of the restoration. [Correction added on 11 September 2025, after first online publication: The previous paragraph has been updated to replace erroneous text.]

Soft tissue ridge augmentation is an effective periodontal surgery procedure to correct ridge defects for esthetic enhancement. Common techniques include connective tissue grafts, roll pedicle grafts, interpositional graft techniques, onlay grafts, and combined onlay‐interpositional grafts [21]. The present defect involved both horizontal and vertical loss of ridge structure, making it particularly challenging to treat, as it necessitates both horizontal and vertical augmentation [40]. Due to several advantages, a SCTG is commonly chosen by clinicians to treat soft tissue defects or gingival recessions [41]. A SCTG, typically harvested from the palate, consists of a dense connective tissue layer without the overlying epithelium [25, 42]. First described by Langer [25], it is a standard technique, offering predictable and reproducible outcomes, which effectively increases soft tissue volume in areas needing both width and thickness enhancement [43]. A SCTG is rich in collagen fibers, fibroblasts, and blood vessels, providing an ideal substrate for soft tissue augmentation and integration at the recipient site. Unlike a full‐thickness graft, the SCTG does not include a keratinized epithelial component, allowing it to be completely submerged at the recipient site for optimal revascularization and healing [25, 44]. Furthermore, a SCTG provides a more natural soft tissue color match, as it is placed beneath the existing gingiva tissue [45]. Due to these advantages, a SCTG sourced from the maxillary tuberosity was chosen to treat the defect in the present case report.

A challenge with RBFDPs is site development, even after sufficient volume has been restored [11]. Acrylic partial dentures serve as effective provisional restorations for RBFDPs due to their ease of removal, replacement, and adjustment. When properly adapted to the proximal teeth, they ensure stable retention and resist vertical displacement. To enhance stability and prevent vertical movement, the removable partial denture should incorporate well‐designed acrylic rests on adjacent teeth [46]. While various techniques exist for pontic side development, most involve an initial perforation of the residual ridge, followed by progressive side modification through the incremental addition of acrylic or composite over multiple clinical visits (typically one to three) [24]. The initial perforation of the soft tissue may be created using a spike‐ or spade‐shaped form, serving as a foundation for further side development. Throughout this process, the spatial relationship between the intaglio surface of the pontic and the underlaying bone must be carefully managed, ensuring a soft tissue thickness of approximately 1–2 mm [47].

Unlike a traditional pontic design, which emphasizes the importance of pressure‐free contact over a small area, an ovate pontic applies gentle pressure to a larger area of the underlying soft tissue [48, 49]. This has the effect of displacing tissue laterally, idealizing the form of the site and creating an emergence profile that imitates that of a natural tooth. When the alveolar ridge is appropriately pretreated beforehand, this design has been shown to yield highly esthetic results [24]. The modified ovate pontic design features a flatter contour with reduced labiolingual thickness compared to the traditional design, allowing excellent esthetic and a more precise replication of the natural tooth emergence profile [50]. Additionally, this technique offers improved cleaning and free gingival margins, while minimizing “black triangles” [50, 51].

## 4 Conclusion

Cantilever zirconia ceramic resin‐bonded fixed dental prostheses combined with soft tissue augmentation present an effective and minimally invasive approach for replacing missing maxillary lateral incisors, offering an alternative to implant therapy. The presented case demonstrates that precise prosthetic planning, tissue management, and appropriate bonding techniques contribute to excellent long‐term functional and esthetic outcomes. Pontic development using an acrylic removable partial denture provides a practical and successful method for site preparation and provisionalization.

## Conflicts of Interest

The authors declare no conflicts of interest.

## Data Availability

Data sharing is not applicable to this article as no new data were created or analyzed in this study.

## References

[1] N. U. Zitzmann , M. Özcan , S. S. Scherrer , J. M. Bühler , R. Weiger , and G. Krastl , “Resin‐Bonded Restorations: A Strategy for Managing Anterior Tooth Loss in Adolescence,” Journal of Prosthetic Dentistry 113, no. 4 (2015): 270–276. 25702966 10.1016/j.prosdent.2014.09.028

[2] S. Nuvvula , V. K. Chava , and S. Nuvvula , “Primary Culprit for Tooth Loss!!,” Journal of the Indian Society of Periodontology 20, no. 2 (2016): 222–224. 10.4103/0972-124X.170852 PMC4847475 27143841

[3] S. Robinson and M. F. Chan , “New Teeth From Old: Treatment Options for Retained Primary Teeth,” British Dental Journal 207, no. 7 (2009): 315–320. 19816477 10.1038/sj.bdj.2009.855

[4] V. Rakhshan , “Congenitally Missing Teeth (Hypodontia): A Review of the Literature Concerning the Etiology, Prevalence, Risk Factors, Patterns and Treatment,” Dental Research Journal 12, no. 1 (2015): 1–13. 25709668 10.4103/1735-3327.150286 PMC4336964

[5] M. Kern , N. Passia , M. Sasse , and C. Yazigi , “Ten‐Year Outcome of Zirconia Ceramic Cantilever Resin‐Bonded Fixed Dental Prostheses and the Influence of the Reasons for Missing Incisors,” Journal of Dentistry 65 (2017): 51–55. 28688950 10.1016/j.jdent.2017.07.003

[6] H. Terheyden and F. Wüsthoff , “Occlusal Rehabilitation in Patients With Congenitally Missing Teeth‐Dental Implants, Conventional Prosthetics, Tooth Autotransplants, and Preservation of Deciduous Teeth‐A Systematic Review,” International Journal of Implant Dentistry 1, no. 1 (2015): 30. 27747652 10.1186/s40729-015-0025-z PMC5005685

[7] M. Sasse and M. Kern , “All‐Ceramic Resin‐Bonded Fixed Dental Prostheses: Treatment Planning, Clinical Procedures, and Outcome,” Quintessence International 45, no. 4 (2014): 291–297. 24570997 10.3290/j.qi.a31328

[8] M. M. M. Gresnigt , J. A. Jonker , and S. A. M. van der Made , “The Cantilever Contact‐Point Resin Bonded Bridge; Adhesion 2.0,” Journal of Esthetic and Restorative Dentistry 36, no. 1 (2024): 37–46. 38084818 10.1111/jerd.13179

[9] J. M. Mendes , A. L. G. Bentata , J. de Sá , and A. S. Silva , “Survival Rates of Anterior‐Region Resin‐Bonded Fixed Dental Prostheses: An Integrative Review,” European Journal of Dentistry 15, no. 4 (2021): 788–797. 34428850 10.1055/s-0041-1731587 PMC8630935

[10] M. B. Blatz , G. Chiche , O. Bahat , R. Roblee , C. Coachman , and H. O. Heymann , “Evolution of Aesthetic Dentistry,” Journal of Dental Research 98, no. 12 (2019): 1294–1304. 31633462 10.1177/0022034519875450

[11] N. Naenni , G. Michelotti , W. Z. Lee , I. Sailer , C. H. Hämmerle , and D. S. Thoma , “Resin‐Bonded Fixed Dental Prostheses With Zirconia Ceramic Single Retainers Show High Survival Rates and Minimal Tissue Changes After a Mean of 10 Years of Service,” International Journal of Prosthodontics 33, no. 5 (2020): 503–512. 32956431 10.11607/ijp.6737

[12] M. Kern , “Fifteen‐Year Survival of Anterior All‐Ceramic Cantilever Resin‐Bonded Fixed Dental Prostheses,” Journal of Dentistry 56 (2017): 133–135. 27832968 10.1016/j.jdent.2016.11.003

[13] S. Habibzadeh , F. Khamisi , S. A. Mosaddad , G. V. O. Fernandes , and A. Heboyan , “Full‐Ceramic Resin‐Bonded Fixed Dental Prostheses: A Systematic Review,” Journal of Applied Biomaterials & Functional Materials 22 (2024): 22808000241250118. 38706266 10.1177/22808000241250118

[14] A. S. A. Al‐Bermani , N. P. Quigley , and W. N. Ha , “Do Zirconia Single‐Retainer Resin‐Bonded Fixed Dental Prostheses Present a Viable Treatment Option for the Replacement of Missing Anterior Teeth? A Systematic Review and Meta‐Analysis,” Journal of Prosthetic Dentistry 130, no. 4 (2023): 533–542. 34893319 10.1016/j.prosdent.2021.10.015

[15] I. A. Alraheam , C. N. Ngoc , C. A. Wiesen , and T. E. Donovan , “Five‐Year Success Rate of Resin‐Bonded Fixed Partial Dentures: A Systematic Review,” Journal of Esthetic and Restorative Dentistry 31, no. 1 (2019): 40–50. 30302909 10.1111/jerd.12431

[16] M. B. Blatz , M. Vonderheide , and J. Conejo , “The Effect of Resin Bonding on Long‐Term Success of High‐Strength Ceramics,” Journal of Dental Research 97, no. 2 (2018): 132–139. 28876966 10.1177/0022034517729134 PMC6429574

[17] M. B. Blatz , A. Sadan , and M. Kern , “Resin‐Ceramic Bonding: A Review of the Literature,” Journal of Prosthetic Dentistry 89, no. 3 (2003): 268–274. 12644802 10.1067/mpr.2003.50

[18] N. P. Quigley , D. S. S. Loo , C. Choy , and W. N. Ha , “Clinical Efficacy of Methods for Bonding to Zirconia: A Systematic Review,” Journal of Prosthetic Dentistry 125, no. 2 (2021): 231–240. 32115220 10.1016/j.prosdent.2019.12.017

[19] C. A. Jurado , A. Tsujimoto , H. Watanabe , et al., “Chair‐Side CAD/CAM Fabrication of a Single‐Retainer Resin Bonded Fixed Dental Prosthesis: A Case Report,” Restorative Dentistry and Endodontics 45, no. 2 (2020): e15. 32483533 10.5395/rde.2020.45.e15 PMC7239681

[20] G. Zucchelli , C. Mazzotti , V. Bentivogli , I. Mounssif , M. Marzadori , and C. Monaco , “The Connective Tissue Platform Technique for Soft Tissue Augmentation,” International Journal of Periodontics and Restorative Dentistry 32, no. 6 (2012): 665–675. 23057056

[21] A. Agarwal and N. D. Gupta , “Alveolar Ridge Augmentation by Connective Tissue Grafting Using a Pouch Method and Modified Connective Tissue Technique: A Prospective Study,” Dental Research Journal 12, no. 6 (2015): 548–553. 26759591 10.4103/1735-3327.170574 PMC4696357

[22] J. S. Seibert , “Reconstruction of Deformed, Partially Edentulous Ridges, Using Full Thickness Onlay Grafts. Part I. Technique and Wound Healing,” Compendium of Continuing Education in Dentistry 4, no. 5 (1983): 437–453. 6578906

[23] R. Gomez‐Meda and J. Esquivel , “Perio‐Prosthodontic Pontic Site Management, Part I: Pontic Designs and Their Current Applications,” Journal of Esthetic and Restorative Dentistry 35, no. 4 (2023): 609–620. 36708252 10.1111/jerd.13023

[24] D. Edelhoff , H. Spiekermann , and M. Yildirim , “A Review of Esthetic Pontic Design Options,” Quintessence International 33, no. 10 (2002): 736–746. 12553617

[25] B. Langer and L. Langer , “Subepithelial Connective Tissue Graft Technique for Root Coverage,” Journal of Periodontology 56, no. 12 (1985): 715–720. 3866056 10.1902/jop.1985.56.12.715

[26] M. Kern , L. Türp , and C. Yazigi , “Long‐Term Outcome of Anterior Cantilever Zirconia Ceramic Resin‐Bonded Fixed Dental Prostheses: Influence of the Pontic Location,” Journal of Prosthetic Dentistry 133, no. 4 (2025): 1017–1023. 40011108 10.1016/j.prosdent.2024.12.032

[27] T. R. Walton , “An up‐To‐15‐Year Comparison of the Survival and Complication Burden of Three‐Unit Tooth‐Supported Fixed Dental Prostheses and Implant‐Supported Single Crowns,” International Journal of Oral & Maxillofacial Implants 30, no. 4 (2015): 851–861. 26252025 10.11607/jomi.4220

[28] S. King , B. Sood , and M. P. Ashley , “Practical Advice for Successful Clinical Treatment With Resin‐Bonded Bridges,” British Dental Journal 235, no. 7 (2023): 503–509. 37828183 10.1038/s41415-023-6332-5 PMC10570136

[29] D. S. Bhusal , S. Khanal , and P. K. Parajuly , “Survival of Resin‐Bonded Fixed Metal‐Ceramic Dental Prostheses Placed in the Anterior Region: A Descriptive Cross‐Sectional Study,” Journal of Nepal Medical Association 59, no. 237 (2021): 494–497. 10.31729/jnma.6500 PMC8673461 34508421

[30] R. Brignardello‐Petersen , “All Zirconia Resin Bonded Fixed Dental Prostheses Replacing Missing Incisors Probably Have a High 10‐Year Survival Rate in Patients Who Are Highly Compliant,” Journal of the American Dental Association 148, no. 12 (2017): e202. 29054277 10.1016/j.adaj.2017.09.023

[31] M. Kern , S. Wolfart , G. Heydecke , S. Witkowski , J. C. Türp , and J. R. Strub , Curriculum Prothetik, Band II, 5 (Auflage, 2022).

[32] J. Chen , H. Cai , X. Ren , L. Suo , X. Pei , and Q. Wan , “A Systematic Review of the Survival and Complication Rates of All‐Ceramic Resin‐Bonded Fixed Dental Prostheses,” Journal of Prosthodontics 27, no. 6 (2018): 535–543. 28985448 10.1111/jopr.12678

[33] L. Mao , M. R. Kaizer , M. Zhao , B. Guo , Y. F. Song , and Y. Zhang , “Graded Ultra‐Translucent Zirconia (5Y‐PSZ) for Strength and Functionalities,” Journal of Dental Research 97, no. 11 (2018): 1222–1228. 29694258 10.1177/0022034518771287 PMC6151910

[34] M. B. Blatz , M. Alvarez , K. Sawyer , and M. Brindis , “How to Bond Zirconia: The APC Concept,” Compendium of Continuing Education in Dentistry 37, no. 9 (2016): 611–617. 27700128

[35] A. Alammar and M. B. Blatz , “The Resin Bond to High‐Translucent Zirconia‐A Systematic Review,” Journal of Esthetic and Restorative Dentistry 34, no. 1 (2022): 117–135. 35072329 10.1111/jerd.12876

[36] A. S. Al‐Amari , M. S. Saleh , A. A. Albadah , et al., “A Comprehensive Review of Techniques for Enhancing Zirconia Bond Strength: Current Approaches and Emerging Innovations,” Cureus 16, no. 10 (2024): e70893. 39497891 10.7759/cureus.70893 PMC11534439

[37] C. D'Alessandro , U. Josic , C. Mazzitelli , et al., “Is Zirconia Surface Etching a Viable Alternative to Airborne Particle Abrasion? A Systematic Review and Meta‐Analysis of In Vitro Studies,” Journal of Dentistry 151 (2024): 105394. 39374733 10.1016/j.jdent.2024.105394

[38] S. J. Kwon , N. C. Lawson , E. E. McLaren , A. H. Nejat , and J. O. Burgess , “Comparison of the Mechanical Properties of Translucent Zirconia and Lithium Disilicate,” Journal of Prosthetic Dentistry 120, no. 1 (2018): 132–137. 29310875 10.1016/j.prosdent.2017.08.004

[39] R. I. Falacho , E. A. Melo , J. A. Marques , J. C. Ramos , F. Guerra , and M. B. Blatz , “Clinical In‐Situ Evaluation of the Effect of Rubber Dam Isolation on Bond Strength to Enamel,” Journal of Esthetic and Restorative Dentistry 35, no. 1 (2023): 48–55. 36325593 10.1111/jerd.12979

[40] S. Pelo , R. Boniello , G. Gasparini , G. Longobardi , and P. F. Amoroso , “Horizontal and Vertical Ridge Augmentation for Implant Placement in the Aesthetic Zone,” International Journal of Oral and Maxillofacial Surgery 36, no. 10 (2007): 944–948. 17629460 10.1016/j.ijom.2007.05.006

[41] K. Kumari , B. Nath , A. Kumar , A. K. Chhabada , R. Kumari , and G. Prakash , “Comparison of Root Coverage by the Subepithelial Connective Tissue Graft With and Without Root Biomodification: A Comprehensive Clinical Study,” Cureus 15, no. 9 (2023): e44758. 37809257 10.7759/cureus.44758 PMC10556792

[42] P. D. Miller, Jr. , “Ridge Augmentation Under Existing Fixed Prosthesis. Simplified Technique,” Journal of Periodontology 57, no. 12 (1986): 742–745. 3467059 10.1902/jop.1986.57.12.742

[43] S. R. Trivedi , N. V. Bhavsar , K. Dulani , and R. Trivedi , “Clinical Evaluation of Subepithelial Connective Tissue Graft and Guided Tissue Regeneration for Treatment of Miller's Class 1 Gingival Recession (Comparative, Split Mouth, Six Months Study),” Journal of Clinical and Experimental Dentistry 6, no. 3 (2014): e218–e224. 25136420 10.4317/jced.51302 PMC4134848

[44] S. A. Saquib , M. Y. S. Bhat , M. A. Javali , S. V. Shamsuddin , and M. A. Kader , “Modified Roll Technique for Soft Tissue Augmentation in Prosthetic Rehabilitation: A Case Report,” Clinical Practice 9, no. 1 (2019): 1110. 10.4081/cp.2019.1110 PMC6434330 30996852

[45] J. S. Seibert , “Treatment of Moderate Localized Alveolar Ridge Defects: Preventive and Reconstructive Concepts in Therapy,” Dental Clinics of North America 37, no. 2 (1993): 265–280. 8477868

[46] K. M. Regish , D. Sharma , and D. R. Prithviraj , “Techniques of Fabrication of Provisional Restoration: An Overview,” International Journal of Dentistry 2011 (2011): 134659. 22013441 10.1155/2011/134659 PMC3195530

[47] A. Mine , M. Fujisawa , S. Miura , et al., “Critical Review About Two Myths in Fixed Dental Prostheses: Full‐Coverage vs. Resin‐Bonded, Non‐Cantilever vs. Cantilever,” Japanese Dental Science Review 57 (2021): 33–38. 33737993 10.1016/j.jdsr.2020.12.002 PMC7946345

[48] R. S. Stein , “Pontic‐Residual Ridge Relationship: A Research Report,” Journal of Prosthetic Dentistry 16, no. 2 (1966): 251–285. 5217111 10.1016/0022-3913(66)90080-1

[49] D. A. Garber and E. S. Rosenberg , “The Edentulous Ridge in Fixed Prosthodontics,” Compendium of Continuing Education in Dentistry 2, no. 4 (1981): 212–223. 6950860

[50] Z. Kaufman and K. S. Paranhos , “Digitally Designed Ovate Pontic as a Predictable Procedure to Improve Accuracy, Hygiene, Esthetics,” Compendium of Continuing Education in Dentistry 43, no. 4 (2022): 226–230. 35380857

[51] S. M. R. Kazmi , Z. Iqbal , M. U. Muneer , S. Riaz , and M. S. Zafar , “Different Pontic Design for Porcelain Fused to Metal Fixed Dental Prosthesis: Contemporary Guidelines and Practice by General Dental Practitioners,” European Journal of Dentistry 12, no. 3 (2018): 375–379. 30147402 10.4103/ejd.ejd_232_18 PMC6089067

